# Heterotopic ossifications in revision reverse shoulder arthroplasty and their impact on long-term outcomes

**DOI:** 10.1186/s13018-025-06312-y

**Published:** 2025-10-08

**Authors:** Raphael Trefzer, Michel Leisner, Mustafa Hariri, Johannes Weishorn, Paul Mick, Matthias Bülhoff

**Affiliations:** Department of Orthopaedics, Heidelberg University Hospital, Schlierbacher Landstraße 200a, 69118 Heidelberg, Germany

**Keywords:** Heterotopic ossifications, Long-term outcomes, Revision reverse shoulder arthroplasty, Clinical relevance

## Abstract

**Background:**

Heterotopic ossifications (HO) are frequently observed after joint replacement surgery and may impair joint mobility and clinical outcomes. The incidence and clinical relevance of HO in revision reverse shoulder arthroplasty (RSA) remain unclear. The purpose of this study was to investigate the (1) incidence of HO and (2) the influence of HO on long-term clinical outcomes in a selected patient cohort who underwent revision RSA.

**Methods:**

We retrospectively identified patients who underwent revision RSA with a minimum follow-up of 5 years. A total of 37 patients (20 female; mean age, 66 years) were examined after a mean follow-up of 10 years. Clinical outcomes included the Constant-Murley Score (CMS) and its age- and sex-adjusted form (aCMS). HO were evaluated on radiographs by two independent raters and graded using a modified Brooker classification. Inter-rater agreement was calculated using Cohen’s Kappa. Statistical comparison of means was conducted using student’s t-test for normally distributed data. Chi square tests were used to assess the incidence between groups. Spearman’s Rho was calculated to investigate correlations between HO grades and clinical outcome.

**Results:**

HO were observed in 28 of 37 patients (75%). Inter-rater agreement was substantial (Cohen’s Kappa = 0.63). The mean aCMS at 10 years was 70.7%; 62% of patients (23/37) reached the patient-acceptable symptomatic state (PASS, defined as aCMS > 61%). HO incidence did not differ between one-stage and two-stage revisions (77% vs. 74%). Higher HO grades were negatively correlated with CMS (r = − 0.412; *p* = 0.013). Patients with HO grade ≥ 2 had significantly lower aCMS values (61.5% vs. 81.6%; *p* = 0.03) and were less likely to reach the PASS (40% vs. 88%; *p* = 0.0003).

**Conclusion:**

HO show a high incidence in revision RSA and are clinically relevant as they are associated with inferior long-term clinical outcomes in grade 2 or higher. Future studies should emphasize on preventive strategies in patients undergoing revision RSA.

**Level of Evidence III:**

Therapeutic study.

## Background

Heterotopic Ossifications (HO) are a radiological phenomenon observed in joint replacement surgery and are mainly described in total hip arthroplasty (THA) [1].

It is assumed that the local tissue reaction triggers periarticular ossification through a proinflammatory and growth-factor induced mechanism [2]. The periarticular bony tissue can lead to limitations in range of motion of the affected joint and therefore compromise functional outcomes [3–5]. In primary reverse shoulder arthroplasty (RSA), HO are observed in around one third of patients and higher grades of HO seem to be associated with inferior short-term functional outcomes [5]. However, literature on this topic is scarce.

In THA, HO occur more often in patients with previous surgery on the affected joint [6, 7]. Until now, no studies exist specifically investigating the clinical relevance of HO in revision RSA in the long-term. Moreover, the influence of HO on long-term clinical outcomes of revision RSA is unknown.

The aim of this study was to assess the incidence of HO and their influence on long-term clinical outcomes in a selected patient cohort who received revision RSA. It was hypothesized that higher grades of HO lead to inferior clinical outcomes.

## Methods

### Patients

In this monocentric study, patients who underwent revision RSA using a 155° neck-shaft angle Grammont style prosthesis with cemented standard stem (Tornier/Stryker Aequalis Reverse II, Memphis, TN, USA) between February 2007 and August 2019 that were documented in a prospective recorded database were included for the clinical and radiological long-term evaluation. Patients who received revision surgery after revision RSA were documented as failed revision RSA but were excluded from the long-term clinical and radiological follow-up evaluation. Patients who were not able to come for the follow-up examination and patients with incomplete clinical or radiological follow-up (pre- and postoperative digital true a. p. and axial views) were also excluded. All patients signed informed consent preoperatively upon entry in the database and at the follow-up assessment. Ethical approval was obtained by a local ethical committee (S305-2007). The study was conducted in accordance with the Helsinki Declaration of 1975, as revised in 2013. Informed consent was obtained from all participating patients.

### Clinical evaluation

Long-term clinical outcomes were assessed using the Constant-Murley Score (CMS) including its subcategories pain, activities of daily life, mobility and abduction strength [8] as well as it’s age- and sex-adjusted form (aCMS) [9]. Isometric abduction force measurements were taken using a Isobex dynamometer (Cursor AG, Bern, Switzerland) at 90° abduction. Active range of motion (ROM) at the follow-up visit was examined using a goniometer.

Patient-reported outcomes (PROs) included the Subjective Shoulder Value (SSV), with a subjective evaluation of the overall condition of the affected shoulder as percentage of a perfectly normal shoulder, and the Simple Shoulder Test (SST), which evaluates the mobility and pain of the shoulder in everyday activity movements.

### Radiological evaluation

Investigation of HO was assessed by two independent raters in standard anteroposterior (true a.p.) and axial radiographs of the shoulder postoperatively within the postoperative course and at the long-term follow-up. A modified Brooker classification was used to grade HO as previously proposed by Verhofste et al. [5, 10, 11]. Grade 1: Bony islands within the periarticular soft tissue. These small bony islands (Grade 1) can morphologically resemble rounded bone fragments and should be distinguished through careful radiographic assessment of their dynamic appearance and localization. Grade 2: Bone spurs with greater than 1 cm distance; Grade 3: Bone spurs with a distance of less than 1 cm; Grade 4: Bony ankylosis of the joint.

Joint reconstruction parameters such as the Lateralized Shoulder Angle (LSA) and the Distalized Shoulder Angle (DSA) were evaluated by the two independent observers as previoulsy described [12].

Further, inferior scapular notching was assessed and classified according to Sirveaux et al. [13].

### Statistical analysis

For statistical analyses SPSS Version 26.0 (IBM®, Armonk, New York, USA) was used. Baseline demographic parameters were analyzed descriptively. Inter-rater reliability was evaluated with Cohen’s Kappa for nominal-/ordinal scaled variables and with Intraclass correlation Coefficient (ICC) for continuous variables. For comparative statistical analyses of preoperative and long-term follow up parameters, paired student’s t-test was used. Comparison of the appearance of HO between two groups in the survival rates was performed using Chi-Square-Test. Correlation analyses of ordinal-scaled radiological classification and clinical outcomes according to CMS, Spearman’s Rho analysis was conducted. Association of factors influencing HO occurrence was investigated using a binary multivariate regression model. Significance level was set to *p* < 0.05.

## Results

A total of 101 patients who underwent revision RSA were identified in the institutional database, out of whom 37 patients met the inclusion criteria and were followed up at a mean of 10.1 years (± 3.9, range 5–17.1). There were 20 women and 17 men, the mean age at surgery was 66 years (± 7.9, range 48–77) (Table 1). Over one third of patients (13; 35%) underwent 2-stage revision procedure to RSA, including 10 patients due to periprosthetic joint infection (PJI) and 3 patients due to aseptic loosening. The indications of the other two-thirds of patients undergoing one-stage revision to RSA were secondary rotator cuff defects (12, 32%), glenoid erosion in patients with failed HA (4, 11%), aseptic loosening (4, 11%) and instability (4, 11%).

**Table 1:** Baseline demographic data

Parameter	Mean	SD	n	%
age	65.9	7.9		
Follow-up [months]	120.9	46.3		
Female sex			20	54.1%
septic revision			10	27.0%
2-stage revision			13	35.1%

The inter-observer and intra-observer reliability according to Cohen’s Kappa for the evaluation of HO between two independent raters were substantial (κ = 0.626 and κ = 0.706) [14]. HO were observed in 28 patients (76%) with a mean age of 67 years including 17 women and 11 men (Table 2).

**Table 2:** Patients grouped into Heterotopic Ossification (HO) grades according to the modified Brooker classification

HO grade (Brooker et al.)	n	%	CMS mean (SD)	PASS reached %
0	9	24.3%	62.3 (21.1)	88.9%
1	8	21.6%	61.1 (19.9)	87.5%
2	9	24.3%	51.4 (22.4)	55.6%
3	7	18.9%	48.9 (23.3)	42.9%
4	4	10.8%	27.0 (8.5)	0.0%

The binary multivariate regression model found no association of HO occurrence with the baseline variables sex, indication for revision RSA (septic or aseptic), one-/two stage revision procedure or initial implant before the revision surgery (RSA, HA or TSA) (Table 3). Further, no association of scapular notching and HO was found in univariate regression analysis (R^2^ = 0.064, Coefficient = 0.115 (95% CI = − 0.039 to 0.268), *p* = 0.138).

**Table 3:** Binary regression analysis of factors influencing HO occurrence; R^2^ = 0.085. RSA = Reverse Shoulder Arthroplasty; HA = Shoulder Hemiarthroplasty; TSA = anatomical Total Shoulder Arthoplasty

Variable	Coefficients (95% CI)	SD	*p* value
Intercept	0.509 (− 0.501 to 1.52)	0.496	0.312
Revision diagnose (septic/aseptic)	0.099 (− 0.512 to 0.71)	0.299	0.742
1-stage vs. 2-stage procedure	0.017 (− 0.553 to 0.588)	0.279	0.950
Initial implant (RSA/HA/TSA)	− 0.082 (− 0.32 to 0.156)	0.117	0.488
Sex	0.215 (− 0.11 to 0.54)	0.159	0.188

The mean CMS at 10 years was 53 points (± 23.2) and the mean aCMS was 70.7% (± 28.2) with 23 patients (62%) reaching PASS according to previously calculated cut-off values for revision RSA [15]. For CMS subcategories, mean “pain” was 9.9 points (± 3.8, range 5–15), mean “activity” was 12.2 points (± 4.5, range 6–20), mean “mobility” was 19.7 points (± 9.4, range 4–36), mean “strength” was 5.2 points (± 4.5, range 0–15). Mean SST was 66.9% (± 18.3) and SSV averaged 55.3% (± 22.5).

**Fig. 1:**
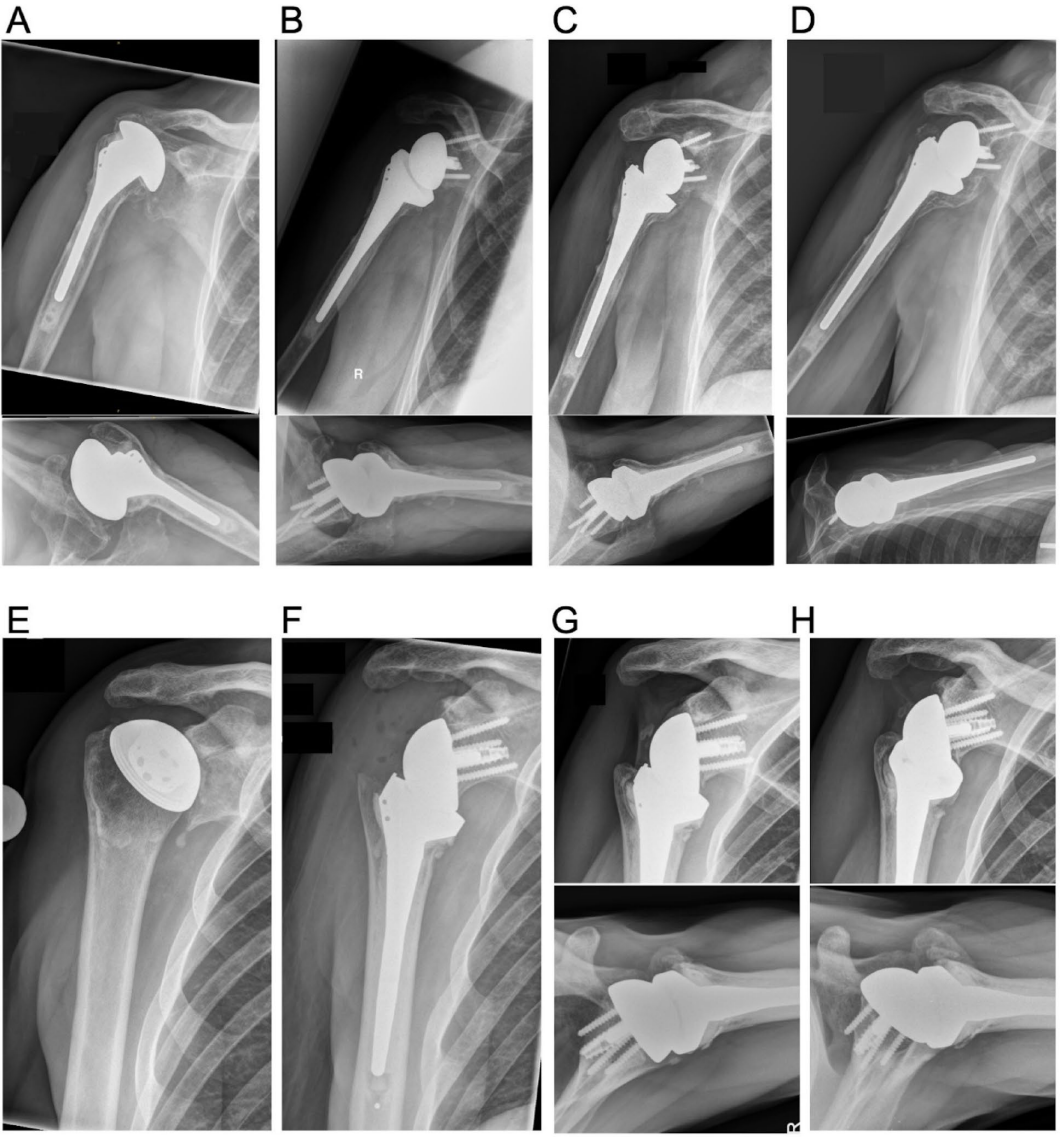
**A** Preoperative true anteroposterior (a. p.) and axial view of the right shoulder of a 62-year-old patient with a cemented stemmed hemiarthroplasty with severe glenoid erosion and massive cranialization of the humeral head due to secondary mass defect of the rotator cuff. **B** Postoperative true a. p. and axial view after one-stage revision to RSA with a cemented stem. **C** 14 months postoperative: Beginning calcification at the medial humeral metaphysis and the inferior scapular neck. **D** Manifest ankylosis due to fusing Heterotopic Ossification at the long-term follow-up at 12 years postoperatively, a Y-view was taken as the axial view was no longer possible. **E** Right shoulder of a 64-year-old male patient with glenoid erosion with a stemless hemiarthroplasty. **F** Postoperative view of the same patient after revision to a cemented standard-stem RSA. **G** Formation of small bony islands in the lateral capsule at 18 months postoperative. **H** The radiographs 9 years postoperatively show a stable course of the small bony islands.

**Fig. 2:**
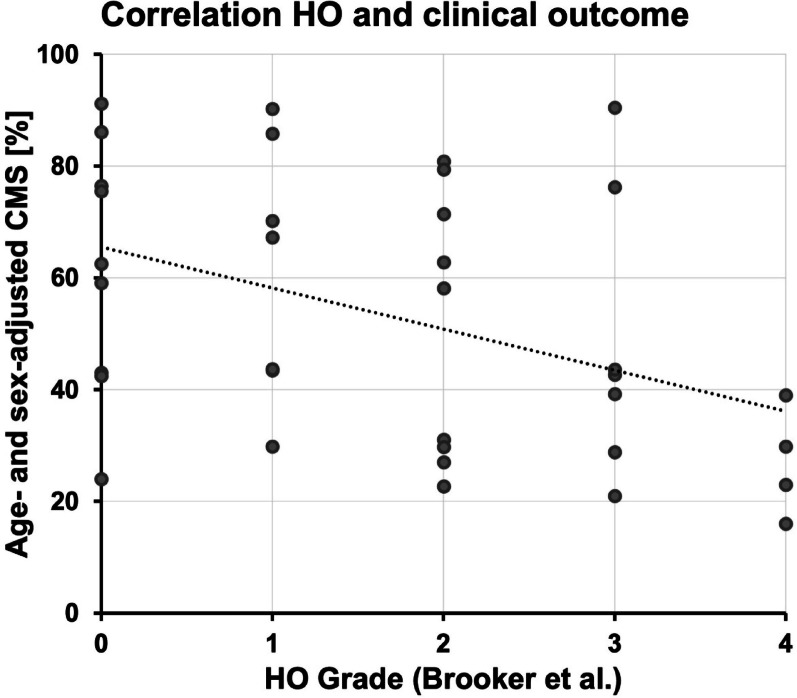
Scatter plot demonstrating the relation between clinical outcome according to sex- and age-adjusted Constant Murley Score (aCMS) and Heterotopic Ossification (HO) grades according to the modified classification by Brooker et al.

**Fig. 3:**
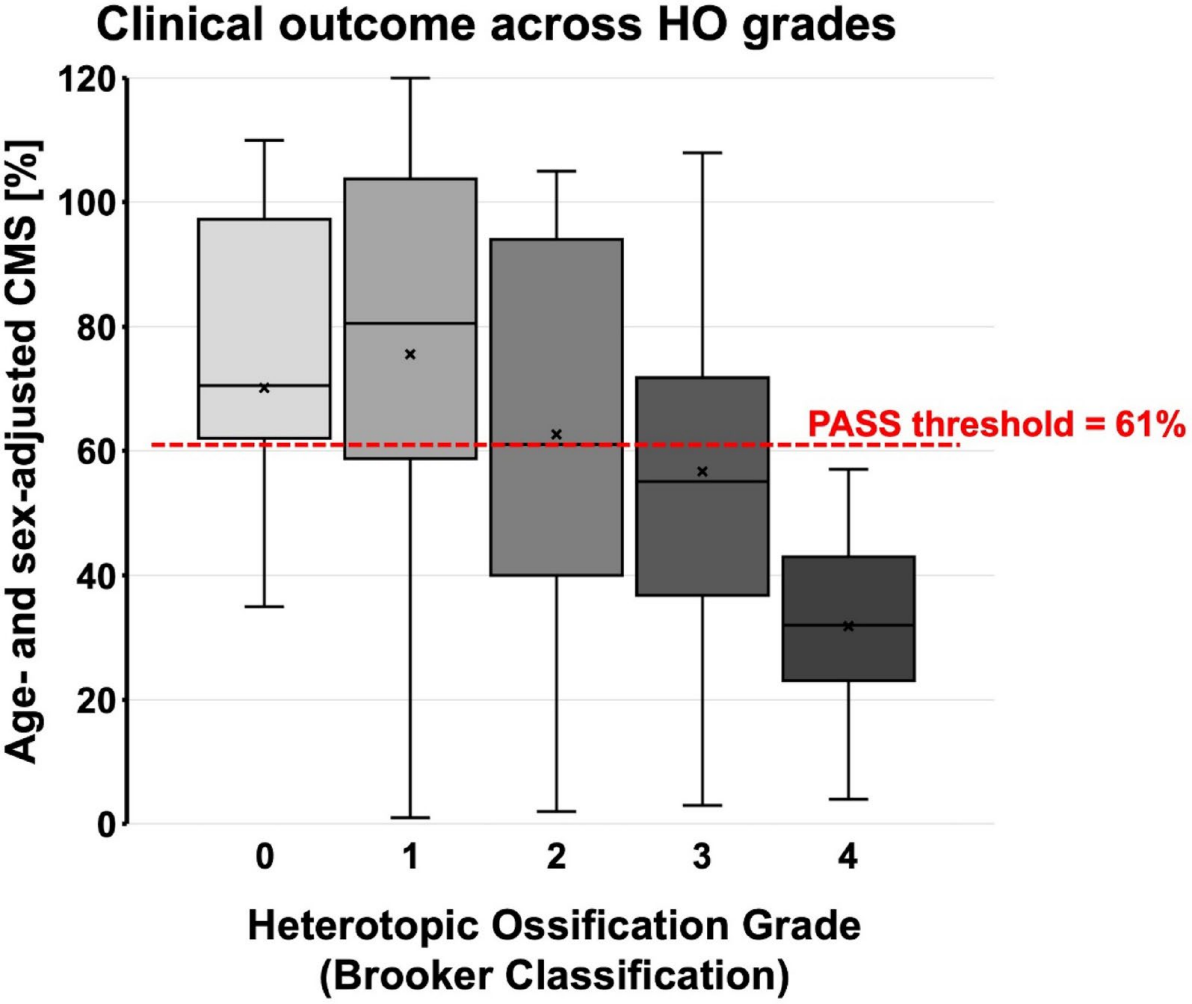
Box-Whiskers plot depicting clinical outcomes with sex- and age-adjusted Constant Murley Score (aCMS) across Heterotopic Ossification (HO) grades according to the modified Brooker Classification. The box marks the interquartile range, the “x” indicates the mean, the band inside the box indicates the median, whiskers indicating minimum and maximum data

A significant linear correlation between long-term clinical outcome according to aCMS and HO grades was found (Spearman’s Rho 0.412, *p* = 0.013) (Fig. 1). Moreover, the mean CMS and the percentage of patients reaching PASS amongst HO grade subgroups decreased in HO grades ≥ 2 (Table 2, Figs. 2, 3).

To further investigate this finding, two groups were established: HO grades 0–1 and HO grades 2–4. Inter-group comparison showed significantly higher mean values of CMS and aCMS reaching MCID and a higher percentage of patients reaching PASS for patients with HO grades 0–1 (88% vs. 40%; *p* = 0.0003) (Table 4).

**Table 4:** Comparison of outcomes between patients grouped into Heterotopic Ossification (HO): no- or low-grade HO (0 or 1) vs. higher-grade HO (2 to 4)

Parameter	HO grade < 2		HO grade ≥ 2		*p* value
	mean	SD	mean	SD	
Age at revision surgery	65.6	8.2	66.1	7.5	0.866
Follow-up [months]	110.5	48.6	129.8	22.8	0.218
CMS [points]	61.7	20.6	45.6	22.8	0.036*
aCMS [%]	81.6	24.5	61.5	27.9	0.030*
ΔCMS pre- vs. postop	43.0	9.8	28.4	21.8	0.100
PASS reached	88.2% (15/17)		40.0% (8/20)		< 0.001*
CMS pain [points]	10.9	3.6	9.0	3.8	0.137
CMS activity [points]	13.3	4.6	11.2	4.2	0.157
CMS mobility [points]	23.4	8.4	16.5	9.2	0.023*
CMS strength [points]	6.4	4.2	4.1	4.6	0.113
SST [%]	71.9	17.8	62.7	18.2	0.132
SSV [%]	62.9	21.8	48.8	21.5	0.054

CMS = Constant-Murly-Score; aCMS = sex- and age-adjusted Constant-Murly-Score; ΔCMS = Difference of CMS points from preoperative to postoperative; PASS = Patient Acceptable Symptomatic State; SST = Simple Shoulder Test; SSV = Subjective Shoulder Value

For the joint reconstruction parameters, a mean LSA of 79.3 degrees (± 11.8) and a mean DSA of 54.5 degrees (13.4) was found. The inter-observer reliability was substantial (ICC = 0.76 for LSA; ICC = 0.65 for DSA). No association with the occurrence of HO (R^2^ = − 0.062; *p* = 0.97 for LSA; *p* = 0.96 for DSA) or HO grades (R^2^ = − 0.062; *p* = 0.93 for LSA; *p* = 0.93 for DSA) was observed in the regression model.

## Discussion

This is the first study reporting about HO in revision RSA in the long-term and specifically investigating the association of HO with PROs. The main findings are (1) that the incidence of HO is high in the selected patient cohort after revision RSA and (2) that HO are clinically relevant as higher grades of HO compromise long-term clinical outcomes.

Recent reviews reported a HO rate in primary shoulder arthroplasty of 26–28% and male sex as a factor being associated with a higher incidence of HO in the short-term [16, 17]. However, no association could be observed between clinical results and HO occurrence in these reviews [16, 17]. In the presented study, no association of the incidence or grade of HO with sex, indication diagnosis for revision surgery, previous primary implant or 1-/2-stage implant exchange procedure was found.

Verhofste et al. reported a HO rate of 29.5% in patients who underwent primary RSA, with lower CMS values compared to patients without HO and no association with scapular notching after a mean follow-up of 36 months [5]. This is congruent with our findings; however, the incidence of HO was substantially higher in our population of revision RSA patients. This could either be explained by repetitive soft tissue damage due to revision surgery or the longer follow-up period. Melis et al. observed similar rates of HO and bony scapular spurs in 75% of primary RSA patients after eight to twelve years [18]. Another study looking specifically at ossifications of the long head of the triceps observed higher rates of HO in revision surgery compared to primary implantation at two years postoperatively [4]. This study provided valuable data on HO incidence in revision RSA with comparable rates but was limited to short-term follow-up and did not assess PROs. Our study expands on these findings by addressing long-term outcomes and their association with PROs.

The rate of HO in THA is also increased in patients with previous surgery on the affected joint [6, 7].

The distance between bony islands and the joint may vary depending on patient positioning and the angle of the X-ray beam. As a result, the modified Brooker classification proposed for grading HO in RSA may be prone to misclassification, particularly due to potential overlap between type II and type III ossifications. However, this limitation also applies to the widely used original Brooker classification for HO after THA, which similarly relies on radiographs affected by limb positioning, especially for axial views. To the best of our knowledge, no more robust or validated alternative classification system currently exists for grading HO in RSA. The subgroup analyses comparing high-grade versus low-grade/no HO in this study are unlikely to be affected by this limitation, as type II and type III lesions were pooled into the same category.

For RSA prosthesis designs with an increased lateral offset, a reduced rate of HO and scapular notching was reported [19–21]. In our cohort, no association between LSA or DSA and HO occurrence nor HO grades could be observed. However, these results should be seen critical due to the small number of patients limiting the statistical power for this specific investigation.

The occurrence of HO has been linked to various etiological factors. Predominantly, the role of bone morphogenetic protein (BMP) as a local inductor and the influence of higher systemic levels of prostaglandine E2 (PGE2) were described [22–24]. Generally, a proinflammatory environment with growth factors and the presence of osteogenic progenitors are assumed to promote HO [2]. However, it’s pathophysiology is only partly understood [25, 26]. HO treatment involves predominantly preventive measures such as NSAID intake or local radiation especially in THA patients [27–29]. A meta-analysis comparing non-selective and selective NSAIDs demonstrated comparable efficacy in the prevention of HO following THA [30]. Naproxen or diclofenac in combination with a proton pump inhibitor were considered appropriate for patients with cardiovascular comorbidities, whereas celecoxib was favored in patients with a history of gastrointestinal disease [31]. Furthermore, a Bayesian network meta-analysis identified celecoxib as associated with the lowest incidence of HO (Brooker grades II–IV) compared with alternative NSAID regimens [32]. In patients suffering from impaired range of motion due to HO, surgical resection can improve outcomes [33]. Our study did not involve specific preventive NSAID intake or surgical treatment for patients with high-grade HO. The high incidence and clinical relevance of HO observed in this specific patient group should prompt future investigations into potential influencing factors and the role of prophylactic measures.

The major limitation of this study is the small number of patients, which compromises the statistical power of explorative analyses regarding associating factors as well as subgroup comparisons. Selection bias cannot be ruled out in the present study, as the analyzed patient group represents only a smaller subcohort of all patients who underwent revision RSA. Consequently, the external validity of the study, especially of the reported incidence of HO, may be limited.

Another limitation is the retrospective study design which only allows for general and associative conclusions. Summarizing, only statements about the descriptive incidence of HO and the negative association between higher-grade HO and long-term clinical outcomes can be made, as the statistical power is too low for assessing influential factors. However, as this is the first study on HO in the context of RSA revision and their impact on long-term clinical outcomes, it represents a unique and valuable contribution to the existing literature.

The perioperative management of patients in this study did not involve a dedicated prophylactic regimen for HO, such as perioperative radiation therapy, or the scheduled use of NSAIDs specifically for HO prevention.

## Conclusions

Considering the high incidence of HO and their clinical relevance in this selected cohort, patients undergoing revision RSA can be seen as a high-risk group.

Future studies should investigate influential factors of HO in revision RSA and standardized prophylactic regimen for HO should be discussed for these high- patients. Further, preventional methods such as the prophylactical intake of nonsteroidal anti-inflammatory drugs (NSAIDs) or preoperative radiation should be the focus of future prospective investigations.

## Data Availability

The data will be available upon reasonable request.
